# The impact of community-based integrated HIV and sexual and reproductive health services for youth on population-level HIV viral load and sexually transmitted infections in Zimbabwe: protocol for the CHIEDZA cluster-randomised trial

**DOI:** 10.12688/wellcomeopenres.17530.2

**Published:** 2023-11-07

**Authors:** Chido Dziva Chikwari, Ethel Dauya, Tsitsi Bandason, Mandikudza Tembo, Constancia Mavodza, Victoria Simms, Constance RS. Mackworth-Young, Tsitsi Apollo, Chris Grundy, Helen Weiss, Katharina Kranzer, Tino Mavimba, Pitchaya Indravudh, Aoife Doyle, Owen Mugurungi, Anna Machiha, Sarah Bernays, Joanna Busza, Bernard Madzima, Fern Terris-Prestholt, Ona McCarthy, Richard Hayes, Suzanna Francis, Rashida A. Ferrand

**Affiliations:** 1 Biomedical Research and Training Institute, Harare, Zimbabwe; 2 MRC International Statistics & Epidemiology Group, Department of Infectious Disease Epidemiology, London School of Hygiene & Tropical Medicine, London, UK; 3 Department of Public Health, Environments and Society, London School of Hygiene & Tropical Medicine, London, UK; 4 Department of Global Health and Development, London School of Hygiene & Tropical Medicine, London, UK; 5 AIDS and TB Unit, Ministry of Health and Child Care, Harare, Zimbabwe; 6 Clinical Research Department, London School of Hygiene & Tropical Medicine, London, UK; 7 Division of Infectious Diseases and Tropical Medicine, University Hospital, LMU Munich, Munich, Germany; 8 Ardent Creative, Harare, Zimbabwe; 9 School of Public Health, University of Sydney, Sydney, Australia; 10 National AIDS Council, Harare, Zimbabwe; 11 Department of Population Health, London School of Hygiene & Tropical Medicine, London, UK

**Keywords:** HIV, youth, community, sexual and reproductive health, Zimbabwe

## Abstract

**Background:**

Youth have poorer HIV-related outcomes when compared to other age-groups. We describe the protocol for a cluster randomised trial (CRT) to evaluate the effectiveness of community-based, integrated HIV and sexual and reproductive health services for youth on HIV outcomes.

**Protocol:**

The CHIEDZA trial is being conducted in three provinces in Zimbabwe, each with eight geographically demarcated areas (clusters) (total 24 clusters) randomised 1:1 to standard of care (existing health services) or to the intervention. The intervention comprises community-based delivery of HIV services including testing, antiretroviral therapy, treatment monitoring and adherence support as well as family planning, syndromic management of sexually transmitted infections (STIs), menstrual health management, condoms and HIV prevention and general health counselling. Youth aged 16–24 years living within intervention clusters are eligible to access CHIEDZA services. A CRT of STI screening (chlamydia, gonorrhoea and trichomoniasis) is nested in two provinces (16 of 24 clusters). The intervention is delivered over a 30-month period by a multidisciplinary team trained and configured to provide high-quality, youth friendly services.

Outcomes will be ascertained through a population-based survey of 18–24-year-olds. The primary outcome is HIV viral load <1000 copies/ml in those living with HIV and proportion who test positive for STIs (for the nested trial). A detailed process and cost evaluation of the trial will be conducted.

**Ethics and Dissemination:**

The trial protocol was approved by the Medical Research Council of Zimbabwe, the Biomedical Research and Training Institute Institutional Review Board and the London School of Hygiene & Tropical Medicine Research Ethics Committee. Results will be submitted to open-access peer-reviewed journals, presented at academic meetings and shared with participating communities and with national and international policy-making bodies.

**Trial Registration:**

https://clinicaltrials.gov/: NCT03719521

## Introduction

Two-thirds of people living with HIV reside in sub-Saharan Africa (SSA). While there has been a general global decline in new HIV infections, these are suboptimal, and this has been much less marked in youth. In 2019, 30% of people newly infected with HIV in eastern and southern Africa were adolescent girls and young women aged 15–24 years ^1^. Compared to other age-groups, youth living with HIV are less likely to be diagnosed and those diagnosed have lower rates of HIV viral suppression once they start antiretroviral therapy (ART) ^2^. HIV testing is a prerequisite for accessing care or prevention services, yet population-based survey data from 2015–2017 in Zimbabwe, Malawi and Zambia showed only 52%, 48% and 45% of youth aged 15–24 years reported ever having an HIV test, respectively ^3– 5^. In these countries, it was estimated that only 40–50% of 15–24-year-olds living with HIV were aware of their HIV status compared with 66–73% in all age groups combined (>15 years). Similarly, HIV viral load suppression among those who reported being on ART ranged from 71–85% among those aged 15–24 but 87–91% among those aged >15 years ^3– 5^. Viral non-suppression is associated with morbidity and with increased risk of onward HIV transmission ^6^.

Youth face unique personal, social, legal, and structural barriers to access HIV services, including judgemental provider attitudes, intense stigma, and families being “gatekeepers” to accessing healthcare ^7, 8^. HIV testing is often not a priority, particularly given the barriers to access.

Worldwide, there remains a large unmet need for sexual and reproductive health (SRH) services among youth, including those who are living with HIV ^9, 10^. The prevalence of curable sexually transmitted infections (STIs) (chlamydia, gonorrhoea, and trichomoniasis) among youth remains very high, and the current approaches for STI management in low- and middle-income settings, i.e., syndromic management has been ineffective in controlling STIs ^11, 12^. The main limitation of syndromic management is that asymptomatic infections, which are the majority, are not treated ^13^. Untreated STIs result in considerable morbidity, including ascending infections, infertility, chronic pelvic pain, and poor birth outcomes. At a public health level, STIs facilitate HIV transmission ^14^.

In recent years, rapid molecular tests that do not require sophisticated laboratory infrastructure have become available, raising the possibility offering screening for STIs in high risk groups such as youth to identify asymptomatic infections ^15^.

This study design is influenced by evidence provided by previous work which can seen here: https://doi.org/10.12688/wellcomeopenres.17531.1 ^16^.

### Study rationale

Youth are a priority group for HIV interventions ^17^, but existing HIV programmes which are vertical and largely facility-based, have not been successful in engaging youth. We hypothesise that services which address the entire HIV cascade (HIV testing, linkage to care and treatment, ART initiation and support to maintain viral suppression) and are community-based may address some of the barriers to access and improve HIV outcomes. Integrating HIV services with SRH services may better address the needs of youth and are likely to lead to higher acceptability and programmatic efficiency ^18^. An important barrier to youth accessing services is healthcare provider attitudes and therefore training and mentorship of healthcare providers to provide youth friendly services is critical to ensure engagement of youth ^18, 19^.

The Community based interventions to improve HIV outcomes in youth: a cluster randomised trial in Zimbabwe (CHIEDZA trial) investigates whether providing an integrated package of HIV and SRH services in community-based settings by trained providers to youth aged 16 – 24 years improves population level HIV viral suppression. The trial embeds another CRT aiming to investigate whether screening for STIs including offering testing, treatment, counselling, and partner treatment (comprehensive management) for chlamydia, gonorrhoea and trichomoniasis reduces population level prevalence of these STIs among youth.

***Study objectives.*** The aim of this trial is to estimate the impact of a comprehensive community-based package of HIV services, integrated with SRH services and general health counselling for youth aged 16–24 years, on population-level HIV viral load.

The objectives are:

1.  To investigate the impact of the intervention on each step of the HIV care cascade (HIV testing, treatment initiation and HIV viral load suppression) among youth at population level ^20^.

2.  To investigate the impact of providing STI screening (chlamydia, gonorrhoea and trichomoniasis) and comprehensive management for those testing positive on population-level prevalence of these STIs among youth.

3.  To measure the uptake of each component of the intervention.

4.  To estimate the cost and cost-effectiveness of the intervention.

5.  To assess the fidelity, feasibility, acceptability, and quality of the intervention as delivered in order to identify likely mechanisms of action for observed outcomes and to inform the factors required for scalability and sustainability.

6.  To estimate population-level prevalence of important health-related risk factors and behaviours among youth.

## Study protocol

### Study design

**Figure 1.**
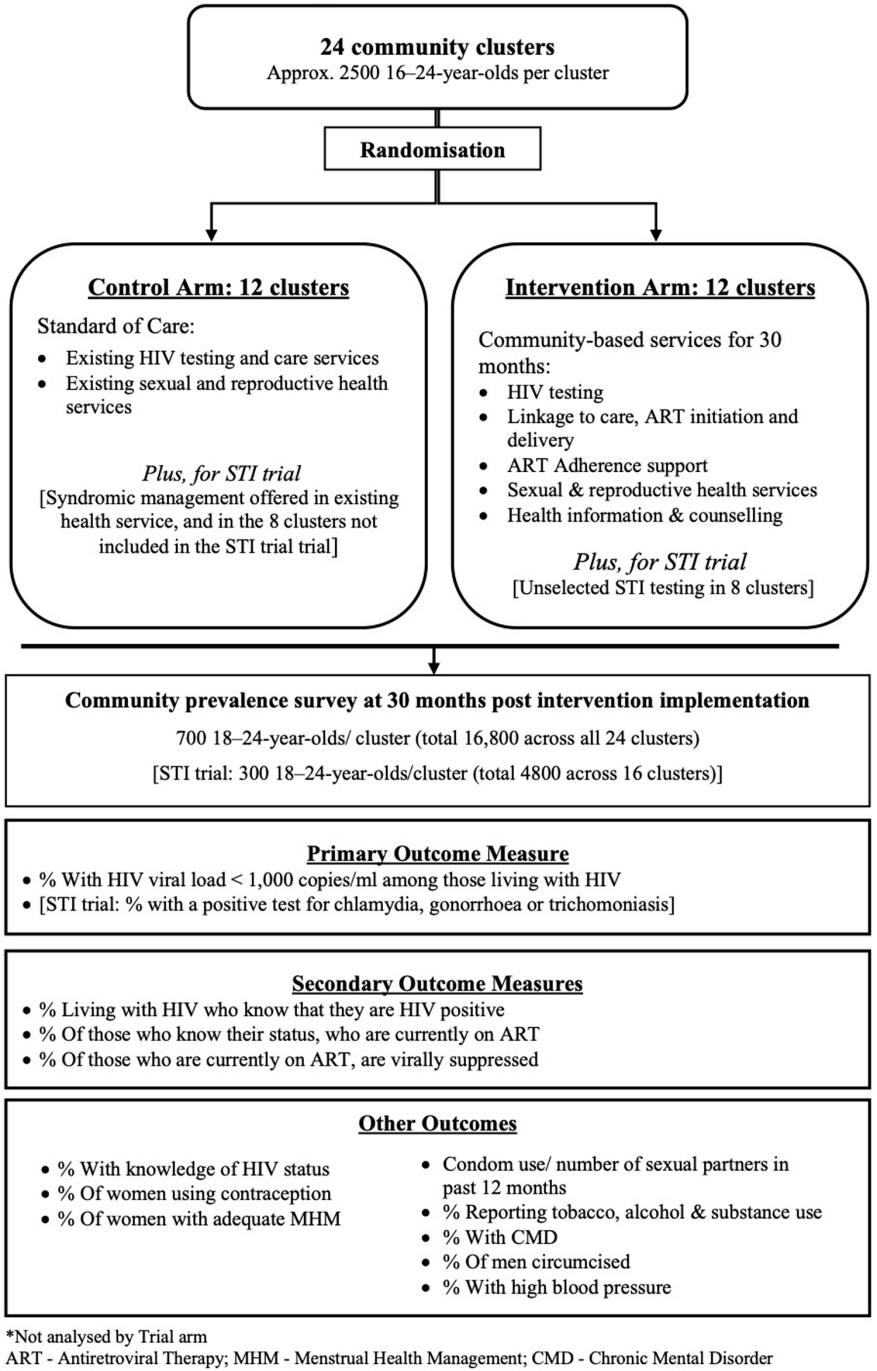
CHIEDZA trial design.

CHIEDZA is a two-arm cluster randomised trial (CRT) ( Figure 1). The standard of care (SOC) is defined as existing HIV and SRH services, which are largely facility-based, siloed and verticalized. For example, within a primary care clinic HIV treatment would be offered by one provider and other services such as family planning would be offered in another part of the clinic by another provider. The standard of care would also include ad-hoc community-based services from various providers, however, no CHIEDZA intervention will be delivered within the control clusters.

A further CRT is embedded within the parent trial to investigate the impact of providing STI screening and comprehensive STI management integrated within the CHIEDZA intervention on population-level prevalence of STIs among youth in two of three CHIEDZA provinces.

### Study setting and population

The trial is being conducted in Zimbabwe, which had an adult (>15 years) HIV prevalence of 12.8% in 2019 ^1^. The trial is being conducted in three provinces, Harare, Bulawayo, and Mashonaland East. Harare is the capital and largest city in Zimbabwe and the population is predominantly of Shona ethnicity; Bulawayo, is the second largest city in the country, situated 437kms from Harare, and is predominantly Ndebele. Mashonaland East province borders Harare and peri-urban settings in this province were selected. In combination, these provinces represent both main ethnic groups in Zimbabwe. Urban and peri-urban settings were selected as population densities in rural areas are low, making the trial unfeasible.

***Definition of study clusters.*** We defined a cluster as a geographically demarcated area with an estimated population of ~2000–4000 youth aged 16–24 years (based on Zimbabwe 2012 Census estimates) that contained a community centre or hall from which the intervention could be delivered ^21^. A cluster had to be serviced by a defined primary care clinic that was not serving another study cluster and was situated within the cluster to ensure integration and collaboration with public-sector services.

Spatial datasets were provided for the study areas by the Zimbabwe Electoral Commission. These included electoral wards (with population numbers), health centres and schools. These data and local field knowledge were used to define a list of possible locations for clusters. These were examined in more detail, overlaying ward populations, clinic and school locations on top of OpenStreetMap data and satellite imagery to show roads, rivers, and buildings ^22^. The clusters were defined by first using electoral ward boundaries to identify areas within the required population range. These areas were then modified to take into account natural breaks e.g. a river or a major road to provide sensible cluster boundaries. Where possible, natural boundaries such as green space or industrial areas were used to form the edge of the cluster to minimise contamination. Satellite imagery and the buildings from OpenStreetMap were used to define an area that was estimated to contain between 2000–4000 youth. Once all the clusters were defined the boundaries and their clinics were exported to Google Earth (.kml format), to enable field teams to check the clinic locations and map key points of interest (POIs). Study staff walked around the cluster, marking any POI onto a printout of the OpenStreetMap data while at the same time storing the location and details as a bookmark in a navigation application (MAPSME) ^23^. The electronic locations were validated against the paper version locations.

**Figure 2.**
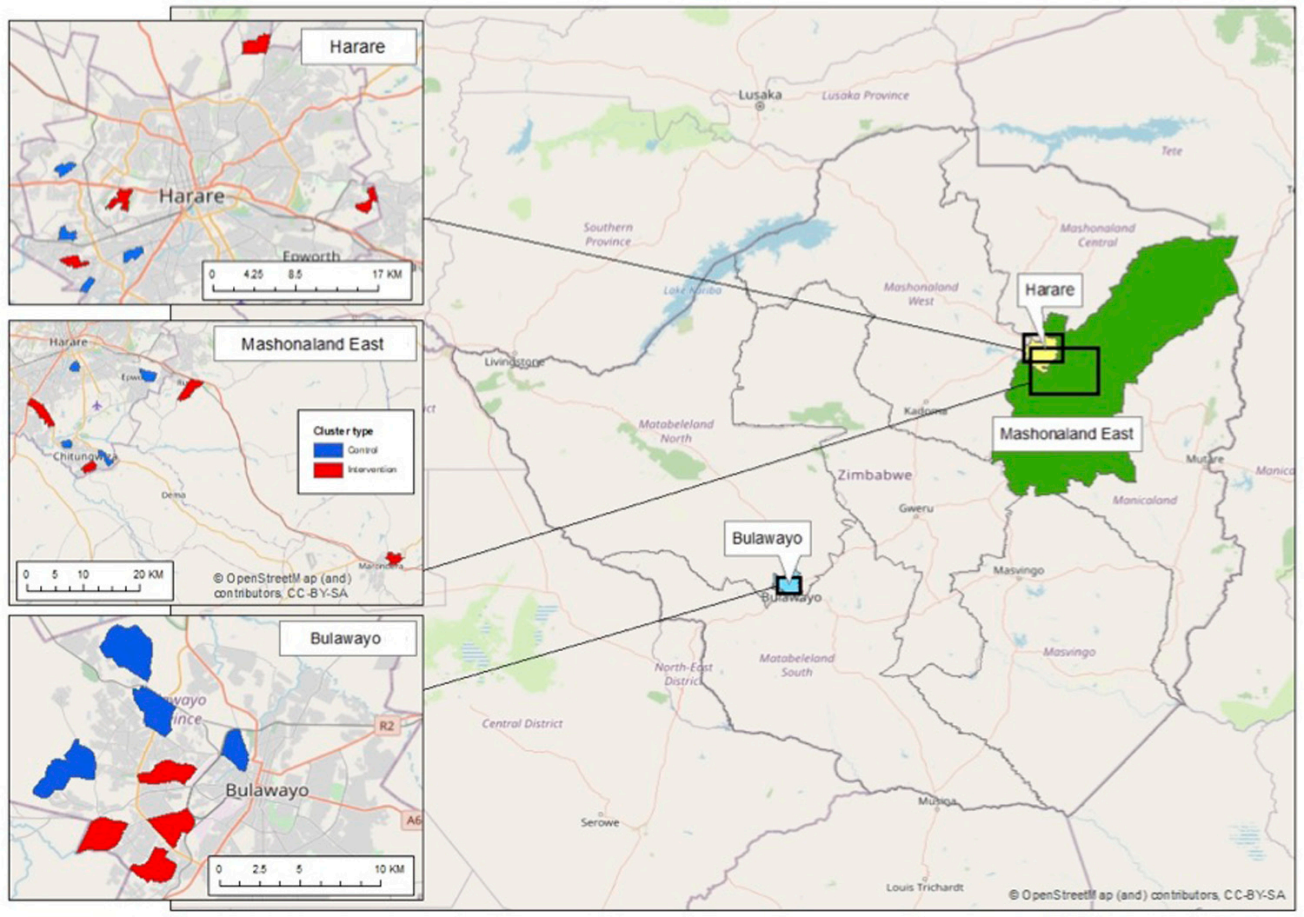
CHIEDZA clusters in the study provinces.

***Randomisation.*** A total of 24 clusters, stratified by province, were randomised in a 1:1 allocation ratio to either the control arm (existing services) or the intervention arm (defined below), so that each province had four intervention and four control clusters ( Figure 2). To maximise transparency and buy-in from stakeholders, public randomisation ceremonies were performed for each province, with representatives of the community, the Ministry of Health and Child Care (MoHCC) and respective City Health or town council health departments. The STI CRT includes clusters in Harare and Bulawayo (total eight intervention and eight control clusters) selected to allow geographic and ethnic variability. The same clusters were intervention clusters for both CHIEDZA and the nested STI CRT.

Youth can receive CHIEDZA services if they are aged between 16–24 years and live within an intervention cluster. Eligibility is assessed by asking individuals who present for CHIEDZA services their age and using the cluster maps with the POIs to identify where individuals live. Youth who are not eligible for CHIEDZA are advised to access services at the nearest health facility.

***Intervention.*** The intervention was designed following formative research that included a literature review, qualitative interviews with relevant stakeholders (n=90) and participatory workshops with youth (n=2) recruited from the study communities. Considering youth opinions and the formative research findings, the research team developed an outline intervention. This outline was presented to youth in participatory workshops, and consensus achieved on the intervention’s content and configuration including the types of services, location of service delivery, types of service providers and the “branding” of the service ( Table 1) *(formative work manuscript submitted with protocol manuscript* ^24^.

**Table 1.** Configuration of the CHIEDZA intervention to provide Youth-Friendly Services.

**Environment**	• Convenient location • Adequate and attractive space where youth can socialise • Comfortable surroundings • Female and male service providers (to avoid a “female-centred” environment) • Providers known by first names • Encouragement of youth-focused “non-health” activities e.g., music, dancing, darts, pool, soccer, film evenings • Outreach - sensitisation of youth and community gatekeepers
**Confidentiality and** ** privacy**	• Multipurpose use venue (so clients not marked out) • Visual and auditory privacy • Names and identifying information not recorded ^a, b^
**Information**	• Paper and electronic IEC materials (posters, videos, manuals) appropriate for various age and literacy levels • Bouquet of SMS messages
**Flexibility**	• Services will be provided from 11am to 7pm and at weekends (e.g., CAPS groups) • Drop-in service delivery (no appointments)
**Youth as equal partners**	• Youth involved in design of space and intervention content and delivery • Youth involved in selection of providers (on interview panel) • Youth Advisory Board for feedback • Youth involved in mobilization and sensitization activities
**Appropriate, quality and** ** affordable services**	• Wide range of services available (see above) • Choice of products where relevant e.g., menstrual management products, contraceptives, • All services and commodities free of charge • High quality products • Trained and mentored providers • System to “red-flag” youth with concerns • Well-established linkages to other services

^a^Biometrics used for recording service uptake and pseudonyms used for managing STI test results ^b^Except where required for clinical care e.g., registration with national HIV care programme IEC - Information, Education and Counselling SMS - Short Message Service CAPS - CHIEDZA Adolescent Peer Support

**Figure 3.**
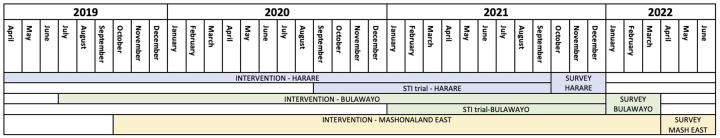
CHIEDZA trial timeline.

The intervention is delivered at multi-purpose community centres or halls within the cluster over a 30-month period. An extension of six months beyond the originally planned 24 months was added to mitigate against the effects of the COVID-19-related lockdowns. Services are provided one day a week (same day every week) in each cluster throughout the intervention period (except for public holidays). The deployment of the intervention across the provinces occurred in phases ( Figure 3).

One intervention team per province is responsible for delivering the intervention to all four intervention clusters. Each team consists of two nurses, four community health workers (CHWs), one counsellor and two youth workers. Youth workers are young people aged below 24 years at the time of recruitment who supported trial activities by welcoming young people to the CHIEDZA sites, screening for eligibility, and responsible for running activities and information sessions. Although the intervention teams are affiliated with the trial, their role is primarily to deliver what is recognized by the World Health Organisation (WHO) as a ‘best practice’ public health interventions ^25^. Hence, the intervention teams are regarded as separate from the ‘research teams’ and the norms and standards governing their activities are those accepted for the implementation of public health interventions rather than those applied to conventional clinical research projects.

The intervention was specifically configured to be “youth friendly” i.e. able to effectively attract youth, meet their needs responsively and retain them in care, as shown in Table 1. In addition, there was a specific focus on intervention providers; the intervention team was selected based on prior experience of working in communities and with youth. A training programme on each of the intervention components was combined with training on provision of youth friendly services, particularly focusing on communication and counselling that is appropriate to age and maturity, and attitudinal training specifically emphasizing respect, confidentiality, non-judgement, and relatability. Ongoing 1–2 weekly debrief meetings that incorporate problem-solving, discussion of complex cases and operational issues ensure that intervention providers are supervised and mentored.

The content of and rationale for the choice of the intervention components is summarised in Table 2. A structured Manual of Operations details intervention activities and guides service delivery (Appendix i). This is updated if any operational procedures are adapted.

**Table 2.** Outline of the CHIEDZA intervention and rationale for choice of components.

Intervention component	Service provided	Eligibility	Rationale
**HIV services covering the whole care cascade**			•Youth have much worse outcomes at each step of the HIV cascade including •Addressing the whole cascade minimises the risk of attrition at each step
**HIV testing**	Choice of: •On-site HIV self-testing using OMT with confirmatory testing of HIV+ve test by provider *or* •Blood-based rapid HIV test by provider + Confirmatory testing of all HIV+ve tests	•All clients of unknown HIV status or having tested HIV-ve >12 months ago or upon request •Testing of partners	•Anticipate high coverage (at least 80%) among youth living with HIV with convenient services in the community, compared to much lower rates through facility-based approach alone ^26^ •Earlier HIV diagnosis
**Linkage to care** ** and ART initiation**	•CD4 count for those testing HIV+ve •TB screening •CrAg testing (if CD4 count <100cells/µl) •Referral for management of serious opportunistic infections (if identified) •Cotrimoxazole prophylaxis •ART initiation on-site •Referral for HIV care at partner PHC *or* to a PHC of client’s choice	•All youth who test HIV+ve or previously tested HIV+ve but not linked to care	•ART initiation at community level kick-starts the linkage to care process ^27^ •Simplification of ART initiation process (without multiple pre-ART initiation visits) improves linkage to and retention in care ^28^ •WHO recommending ART regardless of age and disease stage streamlines ART initiation process ^29^ •ART provision in the community provides flexibility and convenience
**Retention in care** ** & ART adherence**	•ART refills •HIV viral load monitoring ^a^ •Facilitated support groups (CAPS) •Adherence support (defaulter tracing including home visits and SMS messages, specialist counselling)	•Clients with HIV accessing ART through CHIEDZA •Clients with HIV taking ART	•Zimbabwe National Policy recommending ART regardless of age and disease stage streamlines ART initiation process ^29^ •Zimbabwe has a standard decentralised ART delivery approach •Maintaining contact in the community and community-based support encourages retention in care ^30^
**Sexual and reproductive health services**			•Integration of SRH services may increase uptake of HIV testing and other HIV care services •Integration of SRH within HIV care cascade addresses a longstanding gap in programming for youth ^18, 31^
**Menstrual health**	Choice of products ^b^: •Reusable pads •Menstrual cup with a starter pack of disposable pads ^c^ •Underwear •Analgesia •Information & counselling about products	•Female clients	•Information and counselling insufficient without availability of free or affordable commodities ^32^ •Provision of MH products enhances acceptability by community and youth and enhances social marketing ^33^
**Family planning**	•Emergency contraception (“morning after pill”) •Combined oral contraceptive pills •Progesterone-only pills •Pregnancy testing •3-monthly Depo-Provera injections •Referral for post abortion care •IUCD & Implants (depending on availability of partner providers) ^d^	•Female clients •Condoms provided to males and females	•Information and counselling insufficient without availability of free or affordable commodities ^32^
**STI management**	•Syndromic STI management •Partner notification and treatment + *In STI CRT intervention clusters only:* •Unselected STI testing (Trichomonas *vaginalis*, Neisseria *gonorrhoea* and Chlamydia *trachomatis*) ^e^, free treatment and partner notification for those testing positive	•All clients (and treatment of sexual partners)	•High prevalence of STIs in youth in sub-Saharan Africa •STIs increase the risk of HIV transmission •Syndromic management approach both insensitive and non- specific
**Healthy sexual** ** behaviour**	•Risk reduction counselling •Condoms ^f^ •Expedited referral for VMMC	•All clients •All clients •Male clients	
**Age-appropriate** **IEC**	•General health information ^g^ •Bouquet of SMS messages on SRH •Counselling for specific issues including non-health issues •Referral to services ^h^	•All clients	•Improves acceptability and uptake if the intervention is perceived to not have exclusive focus on SRH and HIV and if there is a more holistic approach addressing issues of importance to youth

^a^HIV viral load at 6 months, 12 months and annually thereafter for clients initiating ART and accessing HIV care at CHIEDZA. For clients accessing ART elsewhere but attending CHIEDZA for other services, viral load testing offered opportunistically ^b^Choice of one option (pads or cup) that can be swapped after 3 months ^c^Disposable pads used with a cup initially to provide reassurance that there is no leakage ^d^IUCD and implants delivered by partner providers who come at deliver the services at CHIEDZA ^e^Repeat STI testing offered after 3 months ^f^Flavoured, textured and high quality, with packaging designed to be appealing to youth ^g^Includes an information manual with information on age-appropriate general health and other issues (also available online) ^h^Referral to health and other social and educational services relevant to the issue OMT: Oral mucosal HIV test; TB: Tuberculosis; CrAg: Cryptococcal Antigen; ART: Antiretroviral Therapy; SRH: Sexual and Reproductive Health; IUCD: Intrauterine Contraceptive Device; STI: Sexually Transmitted Infection; VMMC: Voluntary Male Medical Circumcision; IEC: Information, Education and Counselling

***HIV testing and care services.*** HIV testing is conducted according to national guidelines. A mobile-based application (ITHAKA) was used to support HIV self-testing for the first four months of the intervention but discontinued due to logistical challenges. Those who test HIV-positive are offered a choice of being referred to a HIV care clinic of their choice (accompanied by a CHIEDZA team member to the clinic to help facilitate linkage to care) or of accessing care through CHIEDZA. If the latter is selected, the client’s HIV records are maintained at the clinic and clinic data updated by CHIEDZA staff when drugs are collected for supply through CHIEDZA. This ensures that clients remain part of the national HIV programme. HIV treatment is provided according to national guidelines and there are clearly defined referral pathways to a health facility should there be any clinical indications (e.g., severe toxicity, incident symptoms, suspected treatment failure).

Youth living with HIV (regardless of whether they access HIV care through CHIEDZA or not) are offered free HIV viral load testing and enhanced adherence counselling at CHIEDZA if not virally supressed as per Zimbabwe national guidelines as well as membership to the CHIEDZA Adolescent Peer Support (CAPS) groups that are modelled on the existing Community ART Refill Groups (CARGs) implemented in Zimbabwe. CARGs comprise a group of individuals with HIV who meet on a regular basis with one member of the group responsible for collection of ART for other group members. CAPS groups consist of 25–30 youth who meet monthly for semi-structured discussion about issues relevant to youth, social activities and peer support. The facilitator collects drugs for each member of the CAPS group from the local health facility. Those who decline to join CAPS groups can still access their medication through the CHIEDZA service.

***SRH and HIV prevention services.*** Services include advice and information on menstrual health and provision of analgesics and reusable menstrual products, family planning information, counselling, and a choice of short and long-acting contraceptives and pregnancy testing, risk-reduction counselling and condoms and syndromic management of STIs following national guidelines. Easily accessible voluntary medical male circumcision (VMMC) services have been identified for each of the intervention clusters and expedited referral is arranged for clients requesting VMMC. In cases of intimate partner violence (IPV) or sexual assault, youth are referred for to defined services.

Nested STI CRT

Screening for Chlamydia *trachomatis (CT),* Neisseria *gonorrhoea* (NG) and *Trichomonas vaginalis* (TV) is offered to clients in the last 12 months of the intervention in the eight intervention clusters (Harare and Bulawayo). Eligibility includes not having a previous test in the last 3 months. Testing for TV infection is carried out in women only and is performed on self-collected vaginal swabs using a lateral flow assay (Sekisui Diagnostics, Massachusetts, USA) with results available within 15 minutes. Testing for CT and NG infection is performed on urine samples using the GeneXpert platform (Cepheid, California, USA). All clients who take up CT/NG testing can collect results from the CHIEDZA service the following week, but clients who have a positive test result are also contacted by telephone. A minimum of five attempts over three months are made to contact clients with positive CT/NG test results before considering them lost-to-follow-up. For the purposes of follow-up, clients are identified by pseudonyms and no identifying information is collected. All those with positive tests who are contactable and/or return to the CHIEDZA service are provided comprehensive management, including treatment, risk reduction counselling and partner notification slips. Partners are treated free of cost regardless of their age and place of residence.

***Information, education, and counselling.*** Information, education, and counselling (IEC) materials about SRH, HIV and general health issues are available in the form of video clips and a health manual available at the centres and online through the CHIEDZA website. General health counselling is provided, with onward referral to other health service providers for relevant care where appropriate e.g. mental health.

***Mobile based interventions.*** From 18 January 2021, all CHIEDZA clients have been offered the option of registering to receive a series of short evidence-based SMS messages. The messages were developed ^34^ and evaluated ^35– 37^ elsewhere and adapted for the Zimbabwean context though an iterative process with CHIEDZA clients ^38^. The 97 short messages for female recipients (94 for male), delivered over three months and offered in English, Shona and Ndebele contain accurate information about contraception and condom use and promote positive behaviour change ^39^.

***Community sensitisation and mobilisation.*** Service delivery is accompanied by peer outreach to promote CHIEDZA and engage youth to achieve high coverage. Outreach teams consist of 16–24-year-old cluster residents who had previously engaged with CHIEDZA. Following engagement training and social mapping of clusters (which highlighted prime community sensitization locations or “hotspots”), the teams focus their activities around these locations. These include shopping centres, bars, sports centres, boreholes, and community snooker tables. Flyer distribution, information dissemination, and in-field live demonstrations of CHIEDZA products (such as reusable pads, menstrual cups, and condoms) are conducted to educate, generate support and strengthen community engagement. Sensitisation is also conducted door-to-door within the cluster. Mobilisers also escort eligible youth to CHIEDZA centres where requested. During the walk to the centre, mobilisers answer questions and assuage fears. Ad hoc talent shows, modelling, music, drama, and dance competitions are held at community centres to increase their engagement with the intervention. Strategies accommodate the different seasons, school terms and COVID lockdown levels.

***Study outcomes.*** Trial outcomes will be measured at population level. The primary outcome of the CHIEDZA trial is the proportion of survey participants living with HIV who are virally suppressed (defined as having an HIV viral load <1000 copies/ml).

The primary outcome is the product of the following three secondary outcomes among participants living with HIV:

1.  Proportion who are aware of their HIV status

2.  Proportion of those aware of their HIV positive status who are taking ART

3.  Proportion of those on ART who are virally suppressed

Other outcomes among survey participants (regardless of HIV status) are:

1.  Proportion with knowledge of their HIV status

2.  Proportion of females using modern methods of contraception

3.  Proportion of females reporting adequate menstrual management ^40^

The primary outcome of the nested STI trial is the proportion of survey participants who test positive for either NG, CT or TV, ascertained in a randomly selected proportion of participants in the 16 nested STI trial clusters. The secondary outcomes were prevalence of the individual STIs – NG, CT, and TV.

The population-based survey provides an opportunity to understand the prevalence of important health-related risk behaviours and morbidities. These will not be compared by trial arm, and include:

1.  Proportion with symptoms of a common mental disorder (measured using the Shona Symptom Questionnaire-14 with a cut-off of 8) ^41^

2.  Proportions who report tobacco, alcohol, and substance use

3.  Sexual behaviour (including no. of sexual partners in the past year and condom use)

4.  Proportion circumcised (men only)

5.  Proportion who have high blood pressure (Systolic >140mmHg and/or diastolic >90mmHg)

### Ascertainment of study outcomes: endline survey

Study outcomes will be assessed using a population-based cross-sectional survey conducted among 18–24-year-olds living in the study clusters at the end of the intervention period (30 months). The age group was selected to ensure maximum exposure to the intervention.

***Sampling strategy.*** The sampling methodology for the outcome survey combines remote selection methodologies incorporating satellite imagery and traditional random street selection ^42^. The OpenStreetMap road network is checked against satellite imagery to ensure all streets were mapped. All streets within a cluster are manually split into segments within the GIS software (ArcGIS version 10.5) ^43^ either using junctions or features such as school grounds. Segments of streets with no buildings, streets that formed the boundary of the cluster and streets on which the main entrance to buildings is from a different segment are excluded. The average length of segments varies slightly between clusters depending on the density of buildings and streets, ranging from between 100–300m. Each segment is assigned a number and segments are randomly selected. All dwellings in the selected segment will be included in the survey. We estimate between 50–70% of the segments in each cluster will be sampled to reach the required sample size. Maps showing the selected segments and a table with GPS coordinates for the segments will be provided to the survey teams.

***Survey procedures.*** Research teams will visit survey communities before implementation of the survey to sensitize residents about the trial. Following community sensitisation, all households (defined as a person or group of related or unrelated persons who live together in the same dwelling or unit(s) of a dwelling, who acknowledge one adult male or female as head of the household, who share the same housekeeping arrangements, and who are considered a single unit) in each dwelling in the selected street segments will be enumerated. All individuals aged 18–24 years residing in the enumerated households will be eligible to participate. If a potentially eligible individual is not available at the time of enumeration, up to three repeat visits will be made to enrol the individual. 

Following consent, a fingerprint will be collected (see data collection and management section for details) and an interviewer-administered questionnaire will record sociodemographic data, duration of residence and exposure to the intervention. Participants will be asked about knowledge of HIV status, history of HIV testing and care, sexual behaviour, parity, contraceptive use, and menstrual management (females only) and VMMC (males only). In addition, data on smoking and other substance use, mental health, COVID-19 and access to digital technology will be collected. Height, weight, and blood pressure will be measured, and a dried blood spot sample will be collected for anonymised HIV antibody testing and (for those who are HIV antibody positive) HIV viral load testing.

Survey days will be randomised as “STI trial days” for ascertainment of the nested STI trial outcomes. Any participant recruited on a STI day will have a urine sample collected (with additional consent) for STI testing ( *Trichomonas Vaginalis, Neisseria gonorrhoeae and Chlamydia trachomatis*) using the Seegene multiplex real-time PCR (Anyplex II STI Essential Assay, Seegene Inc, Seoul, Korea). Those who test positive for an STI will be followed up for comprehensive STI management. Follow up will be done via telephone (up to three phone calls within two months) and treatment provided for free to trial participants and their sexual partners.

Participants identified with “red flag” conditions (e.g. severe hypertension, experience of violence, severe mental health disorders, alcohol, and drug use disorders) will be counselled and referred to the appropriate health service providers for care.

The survey will occur in phases (by province) following the phased implementation of the intervention ( Figure 3).

### Sample size and statistical analysis

The prevalence survey will recruit 700 youth in each cluster (16 800 total) including 300 within each Harare and Bulawayo cluster (4 800 total) for STI testing. The proportion of youth living with HIV who are virally suppressed (primary outcome), is expected to be 43% in the control arm (60% diagnosed × 84% linked to care × 85% virally suppressed), based on ZIMPHIA estimates ^5^. The community prevalence of HIV in the 18–24-year-old population is estimated to be 3%.

With a coefficient of variation of 0.25, the study will have 80% power to detect a difference of 19% (i.e. 62% prevalence of viral suppression in the intervention arm) and 90% power to detect a difference of 23% (66% prevalence of viral suppression) ^44^. A 66% prevalence of the primary outcome could be achieved by, for example, reaching 80% diagnosis, 91% linkage to care and 91% viral suppression. With a coefficient of variation of 0.3, the study has 80% power to detect a difference of 23% and 90% power to detect a difference of 27%.

For the nested STI trial, with 16 clusters, a sample of 300 youth in each cluster for STI testing will have 90% power to detect an STI prevalence in the intervention arm of 10% compared to 17% in the control arm, with a coefficient of variation of 0.25.

CONSORT guidelines for analysis of CRTs will be followed with CONSERVE guidelines followed to report the trial modifications made as a result of the COVID-19 pandemic ^45^. A flowchart will be created showing the number of clusters eligible, the number allocated to each arm and the number of participants per cluster in the cross-sectional survey. Cluster-level analyses will be used to adjust for between-cluster variability, as recommended for trials with fewer than 15 clusters/arm ^44^. Descriptive analysis will be used to compare cluster-level characteristics of the two arms, with adjustment for variables that are unbalanced between arms (avoiding variables likely to be affected by the intervention) and for stratum.

All outcomes are binary. For each outcome, the risk for each cluster will be calculated, and shown by strata and arm. The mean and SD of the log risk will be used to estimate the geometric mean and associated 95% CI for each arm of the study. Linear regression of the log mean risk on strata and arm will be used to estimate the risk ratio and 95% CI. The approximate variance for the mean risks will be obtained based on the residual mean square from a two-way ANOVA on arm and strata. A 95% CI for this will be calculated from the variance using a t-statistic.

### Data collection and management

For the intervention, each client is registered using a fingerprint using SIMPRINTS software which converts a fingerprint into a Global Unique ID (GUID) (Simprints Technology, Cambridge, UK). SIMPRINTS software is integrated with an Android-based data capture application (Survey CTO Collect) (SurveyCTO, Massachusetts, USA) which is used to record the services accessed by clients on each visit. At the first visit age, sex, date of birth and initials are recorded along with the fingerprint but no identifying information such as name or address is collected. At subsequent visits, the client is identified by fingerprint, which enables providers to check whether the client has accessed the service previously. This allows every client visit and service accessed to be tracked across the intervention period. If biometric identification is not possible or clients decline to provide fingerprints a backup registration process consisting of a unique ID number is allocated to the client. For clients who test HIV-positive, identifying data including name and address and contact details are collected to facilitate linkage to care and register them into the national HIV programme. Details of HIV care including ART prescribed, monitoring test results, and any incident clinical events are recorded on national HIV programme patient records. Data are imported into an ACCESS database for cleaning and quality controlled using automated real-time quality checks.

Endline survey data will be collected on electronic tablets using the SURVEYCTO platform. Biometric records obtained from participants in the cross-sectional survey will be matched against biometric records of clients accessing the intervention to assess degree of contamination (the proportion of control cluster survey participants who accessed the intervention) and coverage of the intervention (proportion of intervention cluster survey participants who accessed the intervention).

### Process evaluation

**Figure 4.**
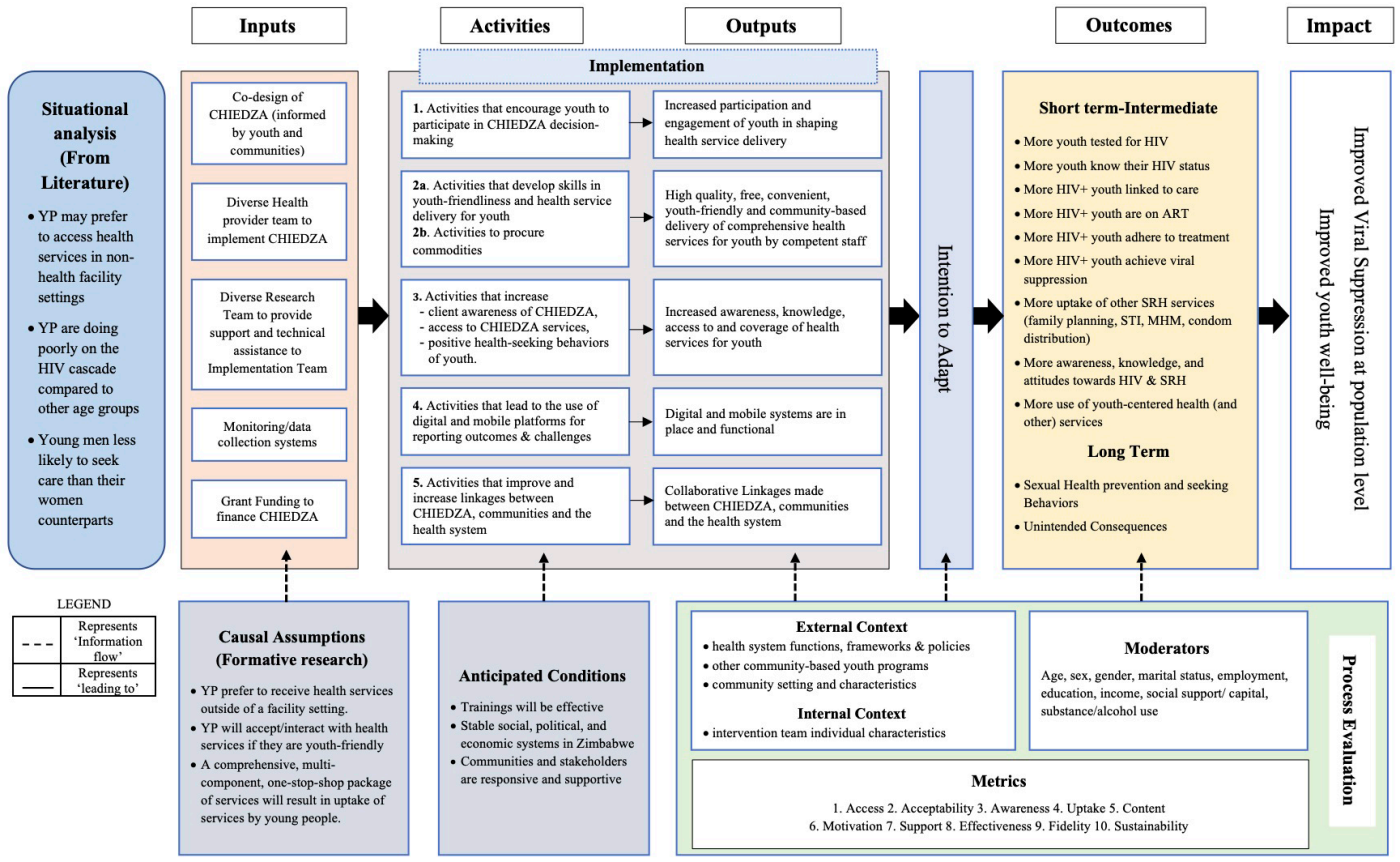
CHIEDZA Logic Model describing the Implementation of the intervention as intended, intentions to adapt, and the anticipated outcomes and Impact.

A detailed mixed methods process evaluation is embedded in the CHIEDZA and nested STI CRT. The process evaluation is based on the MRC Process Evaluation Framework exploring three core functions of the intervention components: i) implementation, ii) mechanisms of impact and iii) context ^46^. An intervention logic model describing the inputs, activities, outputs, outcomes, and intervention impact has been developed ( Figure 4). The situation, causal assumptions and anticipated conditions in the logic model were informed by the formative research ^24^, existing literature and contextual understanding of the study setting. The logic model reflects how the intervention is intended to work. From the outset, the intervention was designed as an adaptative model that would be adjusted in response to implementation and ethical needs, and contextual events during the trial period. The process evaluation therefore had three objectives. First, to assess fidelity, acceptability, feasibility and quality of delivered intervention components, to provide guidance for sustainable and scalable implementation of the intervention. Second, to identify likely mechanisms that produce the trial’s observed outcomes. Third, to offer real-time feedback during the implementation to inform adaptation of the intervention.

The mixed methods approach in the process evaluation includes both sequential explanatory and sequential exploratory approaches ^47, 48^. Qualitative data are collected through 1) non-participant observation of CHIEDZA sites, 2) interviews with both CHIEDZA providers and clients, 3) interviews with youth living in intervention communities and 4) observations of study team meetings. Individual level quantitative data include 1) client demographic information 2) number of attendances over time, 2) uptake of different services at each visit and 3) intervention coverage. As part of the process evaluation, all organisations (health facilities, community-based organisations and non-governmental organisations) providing similar services to those provided by the intervention (e.g. family planning, HIV testing etc) across all clusters are mapped to understand the contribution of these to the outcome. Data are collected longitudinally at multiple time points. Data collection and analysis processes are iterative, and data are triangulated during analysis.

### Economic evaluation

An economic evaluation will be undertaken from the health provider perspective to estimate the incremental cost-effectiveness of the intervention. Primary costing will be undertaken from to estimate the economic costs of all observed resources used to provide the interventions and SOC. Economic costing will be based on global costing guidelines ^49^, and will provide evidence of the total and unit cost of CHIEDZA and STI services and routine HIV and STI services through the SOC. Overall economic costs will include start-up, capital and recurrent costs, including personnel and consumables, and will be estimated using financial costs obtained from expenditure records and economic costs obtained from micro costing. Total costs will be apportioned to usage of service components to estimate unit costs. Incremental cost-effectiveness will be estimated using effect estimates comparing (a) HIV testing and treatment under CHIEDZA and the SOC and (b) STI screening in CHIEDZA and syndromic management under the SOC. In the intervention arm, data on genital symptoms and STI positivity will inform diagnostic accuracy of syndromic management, which will then be applied to calculate the incremental cost-effectiveness of STI screening relative to syndromic management within CHIEDZA.

### Ethics and dissemination

The study protocol has been approved by the Medical Research Council of Zimbabwe (reference number: MRCZ/A/2387), the Institutional Review Board of the Biomedical Research and Training Institute (reference number: AP149/2018) and London School of Hygiene & Tropical Medicine (LSHTM) Research Ethics Committee (reference number: 12063). The trial is registered with the National Library of Medicine (NCT03719521).  

As the intervention components are established public health interventions (e.g. HIV testing, HIV care and provision of SRH services), consent is implied when clients take up intervention activities and specific written consent to participate is not obtained. National guidelines stipulate that those aged 16 years and older can give independent consent to accessing HIV and SRH services. Written, informed consent is obtained for any qualitative interviews conducted as part of the process evaluation. Zimbabwean guidelines stipulate that those aged under 18 years should have parental consent to participate in research but due to the risk of desirability bias affecting responses and the minimal risk associated with participation in interviews, a waiver for parental consent for participants aged 16–18 years was obtained from ethics review bodies.

For the endline survey, eligible individuals are shown a video of the study procedures enacted and narrated by the study team, emphasising that participation is voluntary and that their information will be kept confidential. The video is shown on a tablet with narration in English, Shona or Ndebele and consent is provided electronically on the tablet with a signed paper copy maintained by participants. The video is available online for participants. 

Findings will be presented locally to policy makers and other relevant stakeholders at provincial and national level meetings, and at local academic meetings. Results will also be shared with participating communities and collaborators. International dissemination will be through academic peer-reviewed publications in open-access journals and presentations at international meetings. A study report will be sent to regulatory authorities and a policy brief will be developed to facilitate translation of findings into health policy where appropriate.

### Trial status

At the time of manuscript submission, the intervention is ongoing in two provinces and data collection for the endline survey has begun in one province.

## Discussion

The CHIEDZA trial is the first to investigate the impact of an approach that addresses every step of the HIV cascade on HIV care outcomes among youth. The integration of components is hypothesised to have a synergistic effect because the outcome of each step of the HIV care cascade is conditional on that of the previous step. The trial speaks strongly to WHO recommendations to develop and evaluate client-centred approaches that simplify and adapt HIV services across the cascade and reduce pressure on heavily over-burdened clinics i.e. differentiated service delivery ^50^.

The outcome is of public health importance because viral load suppression reduces HIV-associated morbidity and mortality in individuals. It also reduces risk of HIV transmission with those being virally suppressed considered to be at negligible risk of transmitting HIV to their partners (Undetectable=Untransmissible or “U=U”) ^6, 51, 52^. The trial will assess outcomes at population level, and therefore the outcome is dependent both on the inherent effectiveness of the intervention as well as on its coverage.

Notably, the intervention incorporates comprehensive SRH services, HIV prevention and general health information and counselling. As well as being essential components of HIV service delivery, integration of these services will likely lead to higher acceptability and greater potential for scalability ^14, 29^. Such a model could be adapted to serve as a platform for delivery of services for other chronic conditions e.g. common mental disorders, for which lay counsellor interventions have recently been shown to be highly effective in Zimbabwe and are being scaled-up nationally ^53^.

The nested STI CRT investigates the impact of providing STI screening, free treatment of those testing positive and partner notification on population-level prevalence of STIs. The STI trial will provide data not only on the feasibility and acceptability of STI screening among youth, but also data on the additional yield over and above syndromic management. In addition, the complete STI care cascade including partner notification will be investigated.

The CHIEDZA trial has several strengths: uptake of different intervention components at individual level will be accurately tracked across the intervention, which will provide critical insights into service use patterns. The trial has a biological outcome measure, and the use of biometrics will enable the quantification of intervention coverage and contamination. Taking advantage of the large population-based outcome survey, the project will provide much needed data at population level on health-related behaviours and risk factors for the leading causes of morbidity in youth. The study incorporates a detailed process evaluation and study of cost-effectiveness to inform scalability should it prove effective. We acknowledge several limitations: the study excludes rural settings; the nested STI trial was conducted in two of the three provinces and STI screening was implemented for the last 12 months of the intervention (due to funding constraints), which may not provide sufficient coverage. The COVID-19 pandemic has influenced the trial intervention. Intervention implementation was interrupted for a six-week period in March 2020. Subsequently, adaptations have been made to service provision during lockdowns and curfews. These have included limiting social activities, restricting number of clients and reduced operating hours ^54^. The intervention period was extended by six months to mitigate against some restrictions related to the COVID19 pandemic. The impacts of these adaptations are being investigated in the ongoing process evaluation

In summary, this pragmatic CRT addresses a critical public health issue, and the embedded detailed process evaluation and cost-effectiveness studies will inform scalability and translation into policy and practice should the intervention be effective.

## Data Availability

No data are associated with this article.

## References

[1] UNAIDS: UNAIDS Data.UNAIDS;2020. Reference Source

[2] Slogrove AL Sohn AH : The global epidemiology of adolescents living with HIV: time for more granular data to improve adolescent health outcomes. *Curr Opin HIV AIDS.* 2018;13(3):170–8. 10.1097/COH.0000000000000449 29432227 PMC5929160

[3] Ministry of Health Zambia: Zambia Population-based HIV Impact Assessment (ZAMPHIA) 2016: First Report.Ministry of Health,2017. Reference Source

[4] Ministry of Health Malawi: Malawi Population-based HIV Impact Assessment (MPHIA) 2015-16: First Report.Ministry of Health,2017. Reference Source

[5] Ministry of Helath and Child Care Zimbabwe: Zimbabwe Population-based HIV Impact Assessment (ZIMPHIA) 2015-16: First Report.Harare: Ministry of Health and Child Care;2017. Reference Source

[6] Quinn TC Wawer MJ Sewankambo N : Viral Load and Heterosexual Transmission of Human Immunodeficiency Virus Type 1. Rakai Project Study Group. *N Engl J Med.* 2000;342(13):921–9. 10.1056/NEJM200003303421303 10738050

[7] Chikwari CD Dringus S Ferrand RA : Barriers to, and emerging strategies for, HIV testing among adolescents in sub-Saharan Africa. *Curr Opin HIV AIDS.* 2018;13(3):257–64. 10.1097/COH.0000000000000452 29401121

[8] St Clair-Sullivan N Mwamba C Whetham J : Barriers to HIV care and adherence for young people living with HIV in Zambia and mHealth. *Mhealth.* 2019;5:45. 10.21037/mhealth.2019.09.02 31620472 PMC6789205

[9] Morris JL Rushwan H : Adolescent sexual and reproductive health: The global challenges. *Int J Gynaecol Obstet.* 2015;131 Suppl 1:S40–S2. 10.1016/j.ijgo.2015.02.006 26433504

[10] Shaw D : Access to sexual and reproductive health for young people: bridging the disconnect between rights and reality. *Int J Gynaecol Obstet.* 2009;106(2):132–6. 10.1016/j.ijgo.2009.03.025 19535075

[11] Mayaud P Mabey D : Approaches to the control of sexually transmitted infections in developing countries: old problems and modern challenges. *Sex Transm Infect.* 2004;80(3):174–82. 10.1136/sti.2002.004101 15169997 PMC1744836

[12] Shannon CL Klausner JD : The growing epidemic of sexually transmitted infections in adolescents: a neglected population. *Curr Opin Pediatr.* 2018;30(1):137–43. 10.1097/MOP.0000000000000578 29315111 PMC5856484

[13] Garrett NJ Osman F Maharaj B : Beyond syndromic management: Opportunities for diagnosis-based treatment of sexually transmitted infections in low- and middle-income countries. *PLoS One.* 2018;13(4): e0196209. 10.1371/journal.pone.0196209 29689080 PMC5918163

[14] Barnabas SL Dabee S Passmore JS : Converging epidemics of sexually transmitted infections and bacterial vaginosis in southern African female adolescents at risk of HIV. *Int J STD AIDS.* 2018;29(6):531–9. 10.1177/0956462417740487 29198180

[15] Peeling RW : Applying new technologies for diagnosing sexually transmitted infections in resource-poor settings. *Sex Transm Infect.* 2011;87 Suppl 2(Suppl 2):ii28–30. 10.1136/sti.2010.047647 22110150 PMC3612843

[16] Mackworth-Young C Dringus S Dauya E : Putting youth at the centre: co-design of a community-based intervention to improve HIV outcomes among youth in Zimbabwe [version 1; peer review: 1 approved with reservations]. *Wellcome Open Res.* 2022;7(7):53. 10.12688/wellcomeopenres.17531.1 PMC10804048 38264344

[17] Casale M Carlqvist A Cluver L : Recent Interventions to Improve Retention in HIV Care and Adherence to Antiretroviral Treatment Among Adolescents and Youth: A Systematic Review. *AIDS Patient Care STDS.* 2019;33(6):237–52. 10.1089/apc.2018.0320 31166783 PMC6588099

[18] Denno DM Hoopes AJ Chandra-Mouli V : Effective Strategies to Provide Adolescent Sexual and Reproductive Health Services and to Increase Demand and Community Support. *J Adolesc Health.* 2015;56(1 Suppl):S22–S41. 10.1016/j.jadohealth.2014.09.012 25528977

[19] Ninsiima LR Chiumia IK Ndejjo R : Factors influencing access to and utilisation of youth-friendly sexual and reproductive health services in sub-Saharan Africa: a systematic review. *Reprod Health.* 2021;18(1): 135. 10.1186/s12978-021-01183-y 34176511 PMC8237506

[20] UNAIDS: 90-90-90. An ambitious treatment target to help end the AIDS epidemic.Geneva, Switzerland;2014. Reference Source

[21] Population Census Office: Zimbabwe Population Census Report 2012.Population Census Office;2012. Reference Source

[22] OpenStreetMap.[cited 2021. Reference Source

[23] MAPS.ME.2021. Reference Source

[24] Dringus S Bernays S Doyle A : “Nothing about us without us”: the participation and partnership of youth in co-designing a community-based HIV intervention in Zimbabwe.AIDSImpac; London: aidsimpact.com;2019. Reference Source

[25] Ng E de Colombani P : Framework for Selecting Best Practices in Public Health: A Systematic Literature Review. *J Public Health Res.* 2015;4(3):577. 10.4081/jphr.2015.577 26753159 PMC4693338

[26] Choko AT Desmond N Webb EL : The uptake and accuracy of oral kits for HIV self-testing in high HIV prevalence setting: a cross-sectional feasibility study in Blantyre, Malawi. *PLoS Med.* 2011;8(10): e1001102. 10.1371/journal.pmed.1001102 21990966 PMC3186813

[27] MacPherson P Lalloo DG Webb EL : Effect of optional home initiation of HIV care following HIV self-testing on antiretroviral therapy initiation among adults in Malawi: a randomized clinical trial. *JAMA.* 2014;312(4):372–9. 10.1001/jama.2014.6493 25038356 PMC4118051

[28] Rosen S Maskew M Fox MP : Initiating Antiretroviral Therapy for HIV at a Patient's First Clinic Visit: The RapIT Randomized Controlled Trial. *PLoS Med.* 2016;13(5): e1002015. 10.1371/journal.pmed.1002015 27163694 PMC4862681

[29] Guidelines for Antiretroviral Therapy for the Prevention and Treatment of HIV in Zimbabwe.Zimbabwe: National Medicines and Therapeutics Policy Advisory Committee (NMTPAC) and The AIDS and TB Directorate, Ministry of Health and Child Care, Zimbabwe,2016. Reference Source

[30] Barnabas RV van Rooyen H Tumwesigye E : Initiation of antiretroviral therapy and viral suppression after home HIV testing and counselling in KwaZulu-Natal, South Africa, and Mbarara district, Uganda: a prospective, observational intervention study. *Lancet HIV.* 2014;1(2):e68–e76. 10.1016/S2352-3018(14)70024-4 25601912 PMC4292844

[31] Delany-Moretlwe S Cowan FM Busza J : Providing comprehensive health services for young key populations: needs, barriers and gaps. *J Int AIDS Soc.* 2015;18(2 Suppl 1): 19833. 10.7448/IAS.18.2.19833 25724511 PMC4344539

[32] Cohen DA Farley TA : Social marketing of condoms is great, but we need more free condoms. *Lancet.* 2004;364(9428):13–4. 10.1016/S0140-6736(04)16611-7 15234839

[33] Robinson MN Tansil KA Elder RW : Mass media health communication campaigns combined with health-related product distribution: a community guide systematic review. *Am J Prev Med.* 2014;47(3):360–71. 10.1016/j.amepre.2014.05.034 25145620

[34] McCarthy OL Wazwaz O Osorio Calderon V : Development of an intervention delivered by mobile phone aimed at decreasing unintended pregnancy among young people in three lower middle income countries. *BMC Public Health.* 2018;18(1): 576. 10.1186/s12889-018-5477-7 29716571 PMC5930955

[35] McCarthy OL Aliaga C Torrico Palacios ME : An Intervention Delivered by Mobile Phone Instant Messaging to Increase Acceptability and Use of Effective Contraception Among Young Women in Bolivia: Randomized Controlled Trial. *J Med Internet Res.* 2020;22(6): e14073. 10.2196/14073 32568092 PMC7338928

[36] McCarthy OL Zghayyer H Stavridis A : A randomized controlled trial of an intervention delivered by mobile phone text message to increase the acceptability of effective contraception among young women in Palestine. *Trials.* 2019;20(1): 228. 10.1186/s13063-019-3297-4 31014358 PMC6477750

[37] McCarthy O Ahamed I Kulaeva F : A randomized controlled trial of an intervention delivered by mobile phone app instant messaging to increase the acceptability of effective contraception among young people in Tajikistan. *Reprod Health.* 2018;15(1): 28. 10.1186/s12978-018-0473-z 29433506 PMC5809875

[38] McCarthy O Mavodza C Dziva Chikwari C : Adapting an evidence-based contraceptive behavioural intervention delivered by mobile phone for young people in Zimbabwe.2021. 10.1186/s12913-022-07501-9 PMC8789333 35078457

[39] Kok G Gottlieb NH Peters GJY : A Taxonomy of Behaviour Change Methods: an Intervention Mapping Approach. *Health Psychol Rev.* 2016;10(3):297–312. 10.1080/17437199.2015.1077155 26262912 PMC4975080

[40] Sommer M Sutherland C Chandra-Mouli V : Putting menarche and girls into the global population health agenda. *Reprod Health.* 2015;12(1): 24. 10.1186/s12978-015-0009-8 25889785 PMC4396832

[41] Patel V Simunyu E Gwanzura F : The Shona Symptom Questionnaire: the development of an indigenous measure of common mental disorders in Harare. *Acta Psychiatr Scand.* 1997;95(6):469–75. 10.1111/j.1600-0447.1997.tb10134.x 9242841

[42] Pearson AL Rzotkiewicz A Zwickle A : Using remote, spatial techniques to select a random household sample in a dispersed, semi-nomadic pastoral community: utility for a longitudinal health and demographic surveillance system. *Int J Health Geogr.* 2015;14(1): 33. 10.1186/s12942-015-0026-4 26572873 PMC4647289

[43] ArcGIS Online.2021; [cited 18/11/2021]. Reference Source

[44] Hayes RJ Moulton LH : Cluster Randomised Trials.1st ed. New York,2009;338. 10.1201/9781584888178

[45] Orkin AM Gill PJ Ghersi D : Guidelines for Reporting Trial Protocols and Completed Trials Modified Due to the COVID-19 Pandemic and Other Extenuating Circumstances: The CONSERVE 2021 Statement. *JAMA.* 2021;326(3):257–65. 10.1001/jama.2021.9941 34152382

[46] Moore GF Audrey S Barker M : Process evaluation of complex interventions: Medical Research Council guidance. *BMJ.* 2015;350: h1258. 10.1136/bmj.h1258 25791983 PMC4366184

[47] Ivankova NV Creswell JW Stick SL : Using Mixed-Methods Sequential Explanatory Design: From Theory to Practice. *Field Methods.* 2006;18(1):3–20. 10.1177/1525822X05282260

[48] Creswell JW Plano Clark VL : Designing and conducting mixed methods research.2nd ed. Thousand Oaks, CA: SAGE;2011. Reference Source

[49] Vassall A Sweeney S Kahn J : Reference Case for Estimating the Costs of Global Health Services and Interventions.Global Health Cost Consortium,2017; [September 12, 2017]. Reference Source

[50] Society IA: Differneiated Service Delivery.2021; [cited 2021 18/11]. Reference Source

[51] Attia S Egger M Müller M : Sexual transmission of HIV according to viral load and antiretroviral therapy: systematic review and meta-analysis. *Aids.* 2009;23(11):1397–404. 10.1097/QAD.0b013e32832b7dca 19381076

[52] Cohen MS Chen YQ McCauley M : Prevention of HIV-1 infection with early antiretroviral therapy. *N Engl J Med.* 2011;365(6):493–505. 10.1056/NEJMoa1105243 21767103 PMC3200068

[53] Chibanda D Weiss HA Verhey R : Effect of a Primary Care-Based Psychological Intervention on Symptoms of Common Mental Disorders in Zimbabwe: A Randomized Clinical Trial. *JAMA.* 2016;316(24):2618–26. 10.1001/jama.2016.19102 28027368

[54] Mackworth-Young CRS Mavodza C Nyamwanza R : Other risks don't stop': adapting a youth sexual and reproductive health intervention in Zimbabwe during COVID-19.Sexual and Reproductive Health Matters. In press.2021. Reference Source 10.1080/26410397.2022.2029338 PMC8865116 35192449

