# Supplementary material for: The impact of community-based integrated HIV and sexual and reproductive health services for youth on population-level HIV viral load and sexually transmitted infections in Zimbabwe: protocol for the CHIEDZA cluster-randomised trial
Source: Wellcome Open Res. 2023 Nov 7;7:54. Originally published 2022 Feb 14. [Version 2] doi: 10.12688/wellcomeopenres.17530.2 (PMC10755263; doi:10.12688/wellcomeopenres.17530.2)
Supplement: Peer review history

# Reviewer report: Reviewer response for version 2

**DOI:** 10.21956/wellcomeopenres.22528.r69785

**Published:** 2023-12-28

**Authors:** Sherika Hanley

**Affiliations:** 1 Department of Family Medicine, University of KwaZulu-Natal, Durban, KwaZulu-Natal, South Africa

I am happy with the authors responses.

Is the study design appropriate for the research question?

Partly

Is the rationale for, and objectives of, the study clearly described?

Yes

Are sufficient details of the methods provided to allow replication by others?

Yes

Are the datasets clearly presented in a useable and accessible format?

Yes

Reviewer Expertise:

HIV and NCD prevention, management

I confirm that I have read this submission and believe that I have an appropriate level of expertise to confirm that it is of an acceptable scientific standard.

# Reviewer report: Reviewer response for version 1

**DOI:** 10.21956/wellcomeopenres.19382.r57281

**Published:** 2023-07-17

**Authors:** Sherika Hanley

**Affiliations:** 1 Department of Family Medicine, University of KwaZulu-Natal, Durban, KwaZulu-Natal, South Africa

Thank you for the opportunity to review a well-written protocol of an important study which encompasses critical components of holistic care in youth: a person centered, integrated, community-based approach.

 Although publication is late in the study timeline, there are the benefits of publishing a study protocol close to data analyses including transparency of research processes and allowing for collaboration.

 A few specific comments.

**Overall:**

 Please check and correct for the appropriate use of tense.

**Introduction:**

 Regarding the statement on general global decline in HIV.  UNAIDS Global AIDS Update 2022: "Faltering progress meant that approximately 1.5 million new HIV infections occurred last year—more than 1 million more than the global targets. In too many countries and for too many communities, we now see rising numbers of new HIV infections when we needed to see rapid declines."

 Authors indicate that asymptomatic STIs are not treated with syndromic management. However targeted diagnostic management would probably miss asymptomatic STIs too as there will not be an indication for testing.

**Study design:**

 Are the researchers comparing facility-based care to community-based care?

 Is the integration of services an intervention or delivery strategy?

 It is not clear what existing standard of care services for youth with HIV in the facility entails- is there a "one stop shop" etc.?

 Is BP (and other vital signs) routinely measured under health information as high BP is an outcome? Normal BP for an adolescent is less than 120/80 or 140/90mmHg?

**Study setting and population:**

 Definition of an adult (>15 years) - is this correct?

 Are Harare, Bulawayo and Mashonaland provinces or cities?

 The cluster has to be serviced by a defined primary care clinic. Are these clinics the "control clinics"?

 Please define what youth workers are.

 HIV testing and care services - who collects the ART from the HIV care clinic if client opts for care via CHIEDZA?

 Table 1- info sent by SMS message. What proportion of the youth have mobile devices?

 "Youth as equal partners" - Love this.

 Table 2- Linkage of care and ART initiation- Is there TB prophylaxis provided?

 Is the standard of care "annual" HIV testing/screening in Zimbabwe?

 Besides VL monitoring, are there ALT or CD4 count monitoring?

 Family planning- IUCD and Implants- which sites provided these services? LARC is by far superior.

 Partner notification process- done for all partners?

 Healthy sexual behaviour -male clients are not on the same table row as VMMC.

 Where is the online link for mental health screening?

**Sample size.**

 Is it supposed to state: 80% power- detect a difference of 23% and 90% power to detect a difference of 19%

 Intervention is focused on youth, endline surveys are performed in adults.

 Thank you.

Is the study design appropriate for the research question?

Partly

Is the rationale for, and objectives of, the study clearly described?

Yes

Are sufficient details of the methods provided to allow replication by others?

Yes

Are the datasets clearly presented in a useable and accessible format?

Yes

Reviewer Expertise:

HIV and NCD prevention, management

I confirm that I have read this submission and believe that I have an appropriate level of expertise to confirm that it is of an acceptable scientific standard, however I have significant reservations, as outlined above.

# Response

**Published:** 2023-10-24

**Authors:** Chido Dziva Chikwari

**Affiliations:** London School of Hygiene & Tropical Medicine, UK

**Dear Reviewer, Thank you for the helpful comments to our manuscript. We have now revised the manuscript and below provide responses to your comments. Sincerely, Chido Dziva Chikwari (on behalf of co-authors)**

 Thank you for the opportunity to review a well-written protocol of an important study which encompasses critical components of holistic care in youth: a person centered, integrated, community-based approach.

 Although publication is late in the study timeline, there are the benefits of publishing a study protocol close to data analyses including transparency of research processes and allowing for collaboration.

**The protocol paper was submitted in February 2022 - well before the trial data were analysed. The timeline is related to the review process which we are not in control of.**

 A few specific comments.

**Overall:**

 Please check and correct for the appropriate use of tense.

**We have now reviewed the documents and checked for the appropriate use of tenses. The tense used throughout appropriately reflects the timepoint which this manuscript was written and published.**

**Introduction:**

 Regarding the statement on general global decline in HIV.  UNAIDS Global AIDS Update 2022: "Faltering progress meant that approximately 1.5 million new HIV infections occurred last year—more than 1 million more than the global targets. In too many countries and for too many communities, we now see rising numbers of new HIV infections when we needed to see rapid declines."

**Thank you for noting this point which we agree appropriately reflects the trend in terms of HIV incidence globally. We have now clarified in the text that although there has been some decline in HIV incidence this remains suboptimal.**

 Authors indicate that asymptomatic STIs are not treated with syndromic management. However targeted diagnostic management would probably miss asymptomatic STIs too as there will not be an indication for testing.

**This is an important clarification which we have now corrected throughout the manuscript. Our trial offered STI screening to individuals who were asymptomatic. As such we did not provide targeted diagnostic management and were able to test and treat both individuals with and without symptoms for the three STIs.**

**Study design:**

 Are the researchers comparing facility-based care to community-based care?

**We are comparing community-based care to existing services in communities- these are predominantly facility-based. There may however be vertical, single-service programmes for example a family planning delivery programme and many community-based organisations provide information and education on sexual and reproductive health. However, these would be expected to be equally distributed across the two arms.**

 Is the integration of services an intervention or delivery strategy?

**The intervention is both the integrated services and community-based delivery.**

 It is not clear what existing standard of care services for youth with HIV in the facility entails- is there a "one stop shop" etc.?

**No, there aren’t one-stop shop services – in HIV clinics, HIV treatment and adherence support is offered. Other services are accessed in primary care clinics, by donor-funded vertical programmes or through the government and Ministry of Health. Even the DREAMS programme which also focuses on youth does not offer an integrated services, but beneficiaries are referred to different partners for specific services. The concept of offering integrated HIV and SRH services is the subject of this study. We have clarified this in the methods under the study design.**

 Is BP (and other vital signs) routinely measured under health information as high BP is an outcome? Normal BP for an adolescent is less than 120/80 or 140/90mmHg?

**No it isn’t. The BP is not a trial outcome. Taking advantage of such a large population-based survey, we used it to get population level data on health behaviours and conditions of relevance to youth as such data is scarce in young people. We clarify in the manuscript that these were not trial outcomes.**

**Study setting and population:**

 Definition of an adult (>15 years) - is this correct?

**In the context of HIV prevalence- yes that is correct.**

 Are Harare, Bulawayo and Mashonaland provinces or cities?

**They are provinces. Bulawayo and Harare are also the two largest cities and are also administered as provinces – each with an equivalence of a provincial health directorate.**

 The cluster has to be serviced by a defined primary care clinic. Are these clinics the "control clinics"?

**No, these are not control clinics. These are clinics from where the commodities such as ART was accessed and where clients who registered for HIV care in CHIEDZA would be registered within the HIV programme, where statistics about family planning, and other services would be recorded. This was to ensure that the intervention was not creating parallel reporting structures.**

 Please define what youth workers are.

**Youth workers were young people aged up to 24 years at the time of recruitment. Their specific role was to engage youth who came to the service, and a organise social activities and help foster a youth-friendly, safe environment. They underwent training, were renumerated but did not provide any consultation or specific services other than running information sessions such as condom demonstrations and how to use a menstrual cup. We have now added a definition of youth worker in the manuscript.**

 HIV testing and care services - who collects the ART from the HIV care clinic if client opts for care via CHIEDZA?

**The CHIEDZA nurses did.**

 Table 1- info sent by SMS message. What proportion of the youth have mobile devices?

**We conducted a survey of this prior to the trial implementation. The survey reports on mobile phone ownership among adolescents and youth aged 13-24 years (62.6%). Doyle AM, Bandason T, Dauya E, McHugh G, Grundy C, Dringus S, Dziva Chikwari C, Ferrand RA. Mobile Phone Access and Implications for Digital Health Interventions Among Adolescents and Young Adults in Zimbabwe: Cross-Sectional Survey. JMIR Mhealth Uhealth. 2021 Jan 13;9(1):e21244. doi: 10.2196/21244. PMID: 33439136; PMCID: PMC7840276.**

 "Youth as equal partners" - Love this.

 Table 2- Linkage of care and ART initiation- Is there TB prophylaxis provided?

**TB screening was performed. TB Prophylaxis, while in the guidelines for several years, was not routinely implemented until 2021 when 3HP was rolled out. Prior to 2021, 3 months of isoniazid was recommended in the guidelines but had low rates of offer and uptake and was therefore not routinely implemented.**

 Is the standard of care "annual" HIV testing/screening in Zimbabwe?

**Yes, the current standard of care is annual testing for those who have previously tested HIV negative.**

 Besides VL monitoring, are there ALT or CD4 count monitoring?

**CD4 count was performed at initial assessment. ALT and CD4 count monitoring is not standard of care. It is only performed if clinically indicated.**

 Family planning- IUCD and Implants- which sites provided these services? LARC is by far superior.

**All intervention sites did…however in an effort to ensure that we integrated with what was available already we partnered with an NGO providing family planning to deliver LARCs. Thus, we were reliant on their schedules to deliver LARCs and we recorded when they were present a spart of the process evaluation- and we report on this.**

**We are unclear whether a response to the comment “LARC is by far superior” is required?**

 Partner notification process- done for all partners?

**Yes, this was offered to all clients who had an STI.**

 Healthy sexual behaviour -male clients are not on the same table row as VMMC.

**This has been corrected.**

 Where is the online link for mental health screening?

**We are not clear to what the reviewer is referring to by this comment**

**Sample size.**

 Is it supposed to state: 80% power- detect a difference of 23% and 90% power to detect a difference of 19%.

**We have 90% power with a 23% difference and 80% power with a 19% difference. No changes have been made to the manuscript.**

 Intervention is focused on youth, endline surveys are performed in adults.

**Endline survey performed in individuals aged 18-24yrs. 16–24-year-olds were eligible for services. The age difference is to ensure that all outcome survey participants had had at least 2 years “exposure” to the intervention.**

 Thank you.

# Reviewer report: Reviewer response for version 1

**DOI:** 10.21956/wellcomeopenres.19382.r51157

**Published:** 2022-07-19

**Authors:** Elizabeth Hamlyn

**Affiliations:** 1 King's College Hospital NHS Foundation Trust, London, UK

Well written description of cluster RCT evaluating this important topic.  

 A few minor clarifications are required:

**Introduction:**

- First paragraph "In these countries, it was estimated that only 40–50% of 15–24-year-olds living with HIV were aware of their HIV status compared with 66–73% of those aged >15 years" - it appears comparing the same age groups - do you mean compared to all age groups?  Likewise for following sentence

**Study rationale:**

- What do you mean by existing programmes being "vertical"?

**Study design:**

- It would be useful to describe the current standard of care pathway in more detail - what care do those in control clusters receive?

**Economic evaluation:**

- If successful, how will the study assess whether the intervention reduces long term costs of sequalae of untreated HIV / STI 

Is the study design appropriate for the research question?

Yes

Is the rationale for, and objectives of, the study clearly described?

Yes

Are sufficient details of the methods provided to allow replication by others?

Yes

Are the datasets clearly presented in a useable and accessible format?

Yes

Reviewer Expertise:

HIV infection, adolescent health

I confirm that I have read this submission and believe that I have an appropriate level of expertise to confirm that it is of an acceptable scientific standard.
